# GRANDPARENT CAREGIVING AND EPIGENETIC AGING AMONG MIDLIFE AND OLDER ADULTS IN THE UNITED STATES

**DOI:** 10.1093/geroni/igae098.3132

**Published:** 2024-12-31

**Authors:** Aarti Bhat, Kaitlin Trexberg, Abner Apsley, David Almeida, Idan Shalev

**Affiliations:** The Pennsylvania State University, University Park, Pennsylvania, United States; The Pennsylvania State University, University Park, Pennsylvania, United States; The Pennsylvania State University, University Park, Pennsylvania, United States; Pennsylvania State University, University Park, Pennsylvania, United States; The Pennsylvania State University, University Park, Pennsylvania, United States

## Abstract

In recent decades, grandparents have taken more active caregiving roles, with over 10% of U.S. grandparents raising a grandchild for 6+ months. Role theory postulates grandparent caregivers face unique strain due to complex roles as second-time parents, and physical demandingness of caring for children. Given grandparents are already vulnerable to aging-associated health deterioration, it is important to understand how grandparent caregiving may affect aging processes. Epigenetic aging is an indicator of healthspan and mortality risk that has not been studied in context of grandparent caregiving. DNA methylation patterns at sites associated with markers of aging have led to development of epigenetic “clocks”, which estimate biological age. We examined associations between grandparent caregiving and several epigenetic clocks among a sample of grandparents participating in the Midlife in the United States (MIDUS) study (n=488; mean age=59.41; Black=30.94%; other non-white race=6.56%; female=60.04%). 18.65% of grandparents reported taking on major responsibility for raising grandchild(ren) for 6+ months; among these individuals, mean number of years caring for grandchild(ren) was 5.27 (median=3, range=0-21). Preliminary analyses found, after controlling for sociodemographic characteristics (chronological age was compared against biological age estimates for each clock; race, gender, socioeconomic and marital status were covariates) and BMI (covariate), significant associations between grandparent caregiving and some epigenetic clocks (i.e., DunedinPACE), but not all. Among grandparents who reported providing instrumental care, number of years of care (using median split) was also significantly associated with DunedinPACE. These results indicate prolonged grandparent caregiving may contribute to increased stress and health deterioration at a molecular level.

